# Tablet-Aided BehavioraL intervention EffecT on Self-management skills (TABLETS) for Diabetes

**DOI:** 10.1186/s13063-016-1243-2

**Published:** 2016-03-22

**Authors:** Cheryl P. Lynch, Joni S. Williams, Kenneth J. Ruggiero, Rebecca G. Knapp, Leonard E. Egede

**Affiliations:** Division of General Internal Medicine & Geriatrics, Center for Health Disparities Research, Medical University of South Carolina, 135 Rutledge Avenue, MSC 593, Charleston, SC 29425 USA; Health Equity and Rural Outreach Innovation Center, Ralph H. Johnson VAMC, 109 Bee Street, Charleston, SC 29401 USA; College of Nursing and Department of Psychiatry and Behavioral Sciences, Medical University of South Carolina, 19 Hagood Avenue, Suite 1002, MSC 160, Charleston, SC 29425 USA; Department of Public Health Sciences, Medical University of South Carolina, 135 Cannon Street, MSC 835, Charleston, SC 29425-0835 USA

## Abstract

**Background:**

Multiple randomized controlled trials (RCTs) show that behavioral lifestyle interventions are effective in improving diabetes management and that comprehensive risk factor management improves cardiovascular disease (CVD) outcomes. The role of technology has been gaining strong support as evidence builds of its potential to improve diabetes management; however, evaluation of its impact in minority populations is limited. This study intends to provide early evidence of a theory-driven intervention, Tablet-Aided BehavioraL intervention EffecT on Self-management skills (TABLETS), using real-time videoconferencing for education and skills training. We examine the potential for TABLETS to improve health risk behaviors and reduce CVD risk outcomes among a low-income African American (AA) population with poorly controlled type 2 diabetes.

**Methods:**

The study is a two-arm, pilot controlled trial that randomizes 30 participants to the TABLETS intervention and 30 participants to a usual care group. Blinded outcome assessments will be completed at baseline, 2.5 months (immediate post-intervention), and 6.5 months (follow-up). The TABLETS intervention consists of culturally tailored telephone-delivered diabetes education and skills training delivered via videoconferencing on tablet devices, with two booster sessions delivered via tablet-based videoconferencing at 3 months and 5 months to stimulate ongoing use of the tablet device with access to intervention materials via videoconferencing slides and a manual of supplementary materials. The primary outcomes are physical activity, diet, medication adherence, and self-monitoring behavior, whereas the secondary outcomes are HbA1c, low-density lipoprotein cholesterol (LDL-C), BP, CVD risk, and quality of life.

**Discussion:**

This study provides a unique opportunity to assess the feasibility and efficacy of a theory-driven, tablet-aided behavioral intervention that utilizes real-time videoconferencing technology for education and skills training on self-management behaviors and quality of life among a high-risk, low-income AA population with an uncontrolled dyad or triad of CVD risk factors (diabetes with or without hypertension or hyperlipidemia). The intervention leverages the use of novel technology for education and skill-building to foster improved diabetes self-management. The findings of this study will inform the process of disseminating the intervention to a broader and larger sample of people and can potentially be refined to align with clinical workflows that target a subsample of patients with poor diabetes self-management.

**Trial registration:**

The trial was registered in April 2014 with the United States National Institutes of Health Clinical Trials Registry (ClinicalTrials.gov identifier NCT02128854), available online at: http://clinicaltrials.gov/ct2/show/NCT02128854.

## Background

The southeastern “Diabetes and Stroke Belt” of the US has a high rate of mortality attributed to independent risk factors for cardiovascular disease (CVD) [1, 2], such as diabetes, hypertension, dyslipidemia, and stroke. South Carolina (SC) has one of the highest proportions of African American (AA) residents (28 %) compared to the national prevalence (13 %). In addition, AAs face higher risks of heart disease and stroke-related deaths more often than Whites. AAs are 67 % more likely to have diabetes and had a stroke mortality rate that was 56 % higher than the national average in 2006 [3]. Behavioral risk factors such as poor diet, physical inactivity, and low medication adherence are strongly linked to adverse CVD events. Racial/ethnic differences in CVD risk factors show AAs to have up to a twofold risk of poor control of glycemia, blood pressure, and lipids compared to Whites [4, 5]. Thus, minority populations, AAs in particular, are more likely to suffer poor diabetes and CVD outcomes [6–10]. Updated guidelines for secondary prevention of CVD present compelling evidence that comprehensive risk factor management (pharmacologic and behavioral interventions) improves survival, reduces recurrent events and the need for interventional procedures, and improves quality of life [11]. However, providers face increasingly limited time to focus on individual self-care among those with multiple comorbidities. This study’s approach focuses on enabling self-management behaviors through a multi-component, tablet-aided behavioral intervention that supports self-reliance and behavior change as a potential solution to lowering CVD risk among people with diabetes.

Multiple randomized controlled trials (RCTs) show that behavioral lifestyle interventions are effective in improving diabetes and CVD outcomes [12–16]. A review of three large clinical trials with multifactorial interventions targeting CVD risk among people with diabetes revealed significant improvements in achieving good diabetes self-management behaviors (control of blood pressure [BP], lipids, glycemia [glycosylated hemoglobin A1c, HbA1c], weight, and medication adherence) [17], suggesting that interventions targeting multiple CVD risk factors have a larger impact on CVD risk reduction than single risk factor interventions. Although multiple concurrent and consecutive behavior adjustments can be tremendously difficult to learn and to maintain for patients [18], cross-cutting approaches to multi-morbid conditions will help advance chronic disease management [19]. A systematic review of coping skills training and problem-solving interventions in people with diabetes showed significantly improved diabetes self-management for better metabolic outcomes [20, 21]. Ultimately, problem-solving skills become an integral part of self-management education that helps empower patients to identify problems, adopt strategies to take action, and modify their actions based on different circumstances [22]. The current literature supports the idea that patient-level factors are the key to disease management [23–25] and implementing behavioral interventions with problem-solving and skills training components in high-risk adults is effective in reducing CVD outcomes.

The role of technology has been gaining strong support as evidence builds of its effectiveness for diabetes management [26, 27]. The utility of advanced communication technology in health care is in facilitating medical encounters, increasing access to health care services, and broadening availability of resources, even among underserved populations. Several studies have shown at least equivalent effectiveness of computer-aided [28], telehealth [29, 30], and mobile-enabled interventions on improving diabetes self-care [31] and glycemic control [25, 27]. Another randomized study of 636 patients examined the extent of BP control attained through implementing a multi-component, telephone-delivered, behavioral self-management intervention, home BP monitoring, combined intervention, and usual care [32]. This study showed a significant BP-lowering effect from the combined intervention compared to usual care with a mean of 10 telephone calls (standard deviation, or SD = 3) and a mean call length of 16 minutes (SD = 7); the separate interventions showed no difference. Patients often prefer to rely on these information and communication modalities for access to health care resources and for initiating positive changes in their health behaviors. Yet, the role of technology in minority populations is limited to telehealth interventions that target problem-solving skills [33, 34], which reduces office visits but does not allow consistent daily support to facilitate learning adaptive behaviors for good diabetes self-management (DSM). Studies that focus on AA participants will help to fill a gap in the literature on how behavioral interventions can use technology to better impact self-management of diabetes and other cardiovascular risk factors in this specific subpopulation [25], which generally has poorer glycemic control and higher mortality rates [10]. The proposed study provides early evidence of the theory-driven, Tablet-Aided BehavioraL intervention EffecT on Self-management skills (TABLETS), using real-time videoconferencing for education and skills training, on improving chronic disease self-management (CDSM) behaviors and CVD risk among a high-risk, low-income AA population.

### Rationale

Resources are limited for many AAs in SC, given the disproportionately greater poverty and lower education among AAs compared to Whites [35]. Our pilot RCT will maximize the reach and effect of the intervention and will improve CDSM behaviors (physical activity, diet, medication adherence, self-monitoring) without exceeding available resources [18]. The TABLETS intervention targets control of these modifiable risk behaviors. We use motivational enhancement strategies for low-income AA adults to help them develop a skill set that achieves and maintains good behavioral and clinical outcomes. Potentially successful patient-focused strategies can utilize technology as a tool [31] for personalized learning experiences, and as rapidly interactive and mutually convenient modes of communication between patients and providers [36]. The proposed project will present early evidence of the effectiveness of a multi-component intervention targeting CDSM behaviors using a tablet-based delivery approach for high-risk, low-income AAs. Tablet-aided delivery of the behavioral intervention is best for several reasons: (1) the tablet has a larger visual display to facilitate use among older minority adults — the demographic at highest risk for diabetes, (2) there is greater accessibility of health services and resources for low income populations, and (3) technology-enabled intervention has the added value of convenience, portability, multi-tasking, and personalization, as studies show promising results with small improvements in clinical outcomes among people with diabetes [37, 38]. This project will yield valuable data in preparation for a large-scale RCT to assess whether the lifestyle intervention, in conjunction with tablets as a behavior augmentation tool, has strong potential for promoting long-term behavior change and chronic disease management in underserved populations.

### Study aim and objectives

The primary aim is to examine the efficacy of a multi-component TABLETS intervention on improving behavioral outcomes (physical activity, diet, medication adherence, self-monitoring) in high-risk, low-income AAs with type 2 diabetes using a pilot RCT design. The secondary aims examines the efficacy of the multi-component TABLETS intervention on improving CVD risk profiles and health-related quality of life among high-risk, low-income AAs with type 2 diabetes.

## Methods

The study is a two-arm, pilot RCT with 1:1 randomization of 60 AA participants. Blinded assessments are made at baseline (immediate post-randomization), 2.5 months, and 6.5 months (follow-up). Two booster sessions are delivered via tablet-based videoconferencing at 3 months and 5 months to stimulate ongoing use of the tablet device with access to intervention materials via videoconferencing slides and the web-based portal.

### Location and setting

The study sites are the general internal medicine, endocrine, family medicine, and community care clinics affiliated with the Medical University of South Carolina in Charleston, SC, an urban academic medical center. All screening and assessment activities were held at a research lab. The study team, including the study principal investigator (PI) or co-I, a study coordinator, research assistant (RA), nurse health educator (NHE), and biostatistician, meet frequently to review the study protocol and progress, conduct trainings, and manage unforeseen issues.

### Ethics and trial registration

The study is funded by grant number R03DK098489 from the National Institute of Diabetes and Digestive and Kidney Diseases (NIDDK). The trial is approved by the Institutional Review Board (IRB) of the Medical University of South Carolina.

### Trial population and recruitment

A total of 60 African Americans (AAs) with type 2 diabetes mellitus (T2DM) are being randomized to one of two groups. One study group (*n* = 30) receives the TABLETS intervention, which consists of culturally tailored, telephone-delivered diabetes education and skills training delivered via videoconferencing on tablet devices. The other study group (*n* = 30) receives usual care as provided by their primary care provider (PCP).

Two complementary approaches are used to identify eligible study participants. The first method consists of systematic identification of AA patients with T2DM. After obtaining approval from the IRB, and partial waiver of HIPAA from our local IRB, we use medical or billing records from the prior 6 months to identify (or prescreen) patients with ICD-9 codes for diabetes [250.xx] and hypertension [401.0] or hyperlipidemia [272.0]. The physicians of eligible patients are notified of their patients’ potential eligibility and asked permission to enroll their patients in this study. After consent is obtained from the physicians, letters of invitation on clinic letterhead signed by the patient’s physician are mailed to patients from the study clinics. The letter provides information about the study, explains the study requirements, and clarifies that only patients who meet certain criteria are eligible to participate in the study. The letter includes an addressed and stamped postcard that patients can mail back to indicate interest or lack of interest in participating in the study. In addition, the letter provides a telephone number that interested patients can call to receive detailed information about the study. In the letter, patients are also informed that they will receive a follow-up call in 2 weeks unless they mail back the postcard or call to decline being contacted. Patients who mail back the postcard and express interest or call the provided telephone number receive detailed information about the study. Patients who agree to participate are asked to provide written consent and are scheduled for the initial screening assessment.

The second method consists of referrals from physicians, other clinic staff such as nurses, or patients themselves in response to recruitment flyers for the study. The PI shares the goals of the study and inclusion/exclusion criteria with physicians and clinic staff during clinic administrative meetings. Physicians and clinic staff are asked to refer appropriate patients to the study research assistants. In addition, IRB-approved recruitment flyers are posted in prominent locations in the study clinics.

Regardless of the recruitment pathway, research staff members obtain written informed consent, complete screening for eligibility, and ensure that participants meet criteria for inclusion and participation in the study. The procedure and risks are explained to the patients and the consent form signed as per standard practice. Eligibility has been determined as meeting the following inclusion criteria: self-identified black or African American; age ≥45 years; clinical diagnosis of T2DM (by ICD-9 code of 250.xx) with an HbA1c ≥8 % at screening; clinical diagnosis of hypertension and blood pressure (BP) >140/80 mmHg at the screening visit or clinical diagnosis of hyperlipidemia and low-density lipoprotein cholesterol (LDL-C) >100 mg/dL at screening; able to communicate in English; residence in a 4G cellular service area; and access to a telephone (landline or cell phone). Exclusion criteria include mental confusion or impairment during screening using the validated Mini-Cog instrument [39]; participation in other clinical trials related to the subject area; alcohol or drug abuse/dependence; active psychosis; acute decompensation of chronic conditions that preclude participation; and life expectancy <6 months. Participants who meet eligibility criteria then complete the remainder of the assessment battery and undergo randomization.

### Randomization

**Fig. 1:**
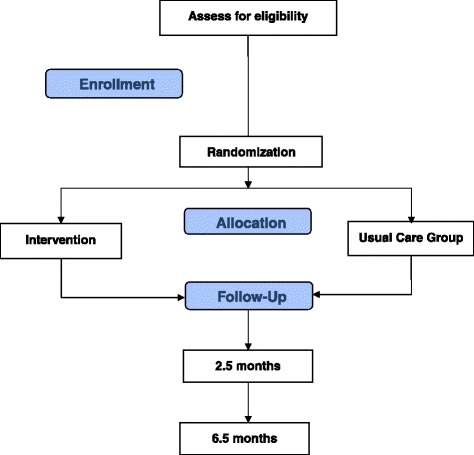
CONSORT flow diagram

All participants are randomly assigned to one of the two study arms (n = 30 per arm) using a permuted block randomization method: (a) TABLETS intervention and (b) usual care (UC). Block size is varied to minimize the likelihood that the blind is broken. The randomization is stratified by baseline HbA1c levels (8–10 % versus >10 %). The RA collects eligibility information and enters the information into the study database via the secured study website. Once eligibility is confirmed, a computer program developed by the biostatistician (RGK) generates the intervention assignment based on the preprogrammed randomization scheme with the order of assignment placed in sealed envelopes until informed consent to participate is signed and eligibility criteria are met. All participants who are randomized are entered into the study database and analyzed according to CONSORT guidelines, as shown in Fig. 1 [40].

### Intervention and control groups

All study participants continued routine clinical visits with their primary care physicians to receive the standard of care.

### TABLETS intervention

The proposed TABLETS intervention builds on the core components of an existing culturally tailored diabetes intervention [41], but it broadens the scope to address multiple CVD risk factors and adds a novel tablet-based delivery mechanism to provide real-time videoconferencing education about DSM behaviors to high-risk, low-income AA adults with diabetes. Intervention participants receive a complimentary manual of documents arranged in specific sections to provide (1) instructions on the use of tablets and biometric measurement devices (glucometer, sphygmomanometer, weight scale) and information about CVD; (2) logs to support self-monitoring of blood glucose, blood pressure, nutrition/dietary plans, and behavior changes; and (3) brief education sheets to enhance behavioral skills for lifestyle change such as tips for safe exercising, practical dietary modifications, reading nutrition labels, and stress management. CVD knowledge/information modules consist of materials developed from a CVD patient education booklet adapted from the Maine Heart Center of Maine Health [42] and supplemented by clinical guidelines to specifically address behavioral risk factors (that is, the four intervention components). Motivation and behavioral skills training modules consist of patient activation (asking questions to providers), patient empowerment (CVD responsibility contracts, flow charts for lab results), and behavioral skills training (self-monitoring, goal-setting). Culturally tailored components have evolved from understanding the food content, social practices, and acceptable forms of physical activity in the southern urban AA culture. In addressing sociocultural and environmental factors [43, 44], it becomes necessary to change or modify usual interventions to the unique needs of rural individuals in order to maximize its effectiveness in changing behaviors such as physical activity, diet, and self-monitoring. Cultural tailoring of the proposed intervention has involved aligning specific components with food preferences in southern cuisine, incorporating social interactions in churches and/or among families and friends, and including physical activities AA participants were willing to engage in over time.

### Usual care group

Participants randomized to the UC group receive usual care for diabetes management as provided by their PCP and generally defined by the American Diabetes Association clinical practice guidelines. When considering clinical relevance and future directions, we decided it was critical to establish that the TABLETS intervention is better than usual care and that results are due primarily to the intervention, since previous studies have established the effectiveness of individual components. Thus, we chose the pilot RCT design to examine whether the effect of the TABLETS intervention exceeds that of UC and the results are not due to time trends or other alternative reasons. The issue of attention control was considered with this group. However, it was decided that comparing the intervention to true usual care, as it reflects current clinical practice during the time of this study, would provide a clearer sense of the intervention’s effectiveness in changing behavior and more accurate estimates of cost outcomes.

### Technology of individual intervention sessions

**Fig. 2:**
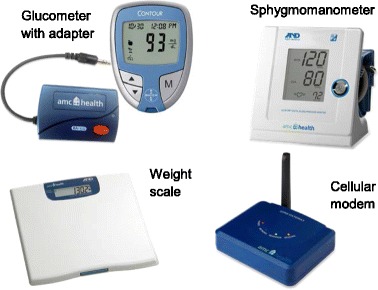
Technology Package of Biometric Measurement Devices with Associated Cellular Modem

Each participant in the intervention group receives one tablet computer and three biometric measurement devices (glucometer with adapter, sphygmomanometer, and weight scale; see figure). The tablet is provided to participants enclosed in a case that allows it to recline on a flat surface and facilitate viewing during weekly videoconference education sessions. In the 2-week run-in phase, participants were instructed to use the tablet device and navigate the internal functionality (use of a predefined set of apps including an email app), check their email to ensure receipt and retrieval of messages sent to them, and use the Internet app to explore information on the web. Participants are allowed to use the Internet on tablets to web browse with restrictions. During videoconferencing, the NHE simultaneously screen shares slides to reinforce major learning points and, when needed, can also use a whiteboard feature to allow a visual for simple mathematical calculations. Participants are asked to use the biometric measurement devices to monitor their blood sugar and blood pressure on at least a daily basis and their weight on a twice-weekly basis. Each of the measurement devices transmits readings wirelessly to a cellular modem (see Fig. 2) that forwards data to a web-based portal database. The NHE accesses the portal prior to each intervention session to review the prior weeks’ readings with participants. Participants are also able and encouraged to access the portal and view their own readings in a tabular or graphical format on the tablet and have the option to do the same on their personally owned computer. In addition, the NHE can enter, save, and push reinforcing or celebratory messages to participants such as “Good job keeping track of your numbers”, “We can work on getting your blood pressure in range”, or “Happy Birthday!”.

### Content of individual intervention sessions

Intervention sessions comprise a multimedia collection of communication tools including (1) interactive videoconferencing segments to deliver education and skills training, (2) supplemental videoconferencing slides, and (3) a whiteboard that provides visual aids aligned with educational materials. Presentations incorporate the participant’s CVD risk factor data and their 10-year CVD risk based on Framingham Risk Score (FRS) [45] estimates, according to seven factors (age, gender, total cholesterol (TC), high-density lipoprotein cholesterol (HDL-C), systolic BP, anti-hypertensive medication use, and smoking status).

#### Session 1

Session 1 is designed to be a face-to-face encounter with the NHE to review study goals and the schedule of study sessions, obtain participant contact information (primary and alternate telephone numbers), establish a schedule for follow-up calls, and receive study materials. For the intervention group, subsequent sessions comprise education and skills training (15 minutes of content delivery followed by 15 minutes of skills training) for interactive learning. The comparator group receives usual care provided by their primary care physician.

#### Sessions 2–9

Each of the eight main intervention sessions falls under one of three areas of focus as described below.

*Information*: The NHE reviews the CVD education booklet with participants and provides visual examples of items such as a sample nutrition label and predefined menu with healthy alternatives. Education is also provided on increasing fruit/vegetable/fiber intake, limiting fat/cholesterol/salt intake, and minimizing added sugars; and building up physical activity to moderate intensity as aligned with guidelines (American Diabetes Association [46], American Heart Association (AHA) Diet and Lifestyle Recommendations [47], AHA/American College of Sports Medicine [48]). Information is provided on medication effects/side effects and strategies for self-monitoring. All education modules are designed to be relevant and person-centered. *Motivation*: Embraces patient activation and empowerment and works toward goals to improve communication skills with health care providers by asking questions at clinic visits about setting goals for A1c, BP, and cholesterol, reviewing monitoring logs, or need to change medications. The NHE is trained to empower patients by providing tools for CVD self-management guided by their “Heart Health Care Package,” which contains a responsibility contract; personal goals sheet; listing of good health practices; self-management kit; flow sheets for lab results/medications; and blood sugar and BP logs. *Behavioral skills*: Aims to equip participants with the skills needed to increase self-efficacy and behavior change through a series of skills training sessions with the NHE, who emphasizes individualized problem-solving. Skills training is based on the four DSM behaviors and review of home monitoring devices (including blood glucose, blood pressure, and pedometer readings) along with medication adherence strategies and tracking data via portal. Guided by participants’ problem areas and preferences, the NHE assists participants in the process of choosing weekly target goals for each behavior over 8 weeks with action plans. During each session the NHE implements strategies learned during motivational enhancement training.

#### Sessions 10–11

Two booster sessions are delivered on tablets at 3 and 5 months post-randomization, during which the NHE reviews goals from action plans, discusses problem-solving, and implements motivational strategies for DSM behaviors.

The NHE participates in routine training by a psychologist (KJR) with expertise in behavioral activation and motivational enhancement. The training involves learning motivational skills through discussion of relevant materials, case-based scenarios, role-playing, and strategic alignment of motivations of study participants. This training facilitates the NHE in developing and refining the skills to empower patients in engaging with their PCPs as well as understanding their numbers and problem-solving for their own self-management using the tools provided in the Heart Health Care Package described above.

Individuals from the study’s intervention group are asked to participate in 30- to 40-minute, semi-structured interviews by telephone to assess usability outcomes for the tablet computer and its associated peripheral measurement devices that help with refinement of the intervention. All interviews are audio-recorded to ensure accuracy of data and for anonymous use of selected verbatim quotes. Therefore, all participants will have acknowledged and agreed through the informed consent process that their participation in interviews requires their consent to be audio-recorded, and they are asked to provide verbal agreement at the time of the interview. After each session, audio recordings are downloaded into a secure, password-protected database developed for this study and later transcribed into a word processing file that is stored on the secure, password-protected web databases developed for this study. Approval from the IRB was obtained prior to audio-recording any interviews.

### Study instruments and data collection schedule

**Fig. 3:**
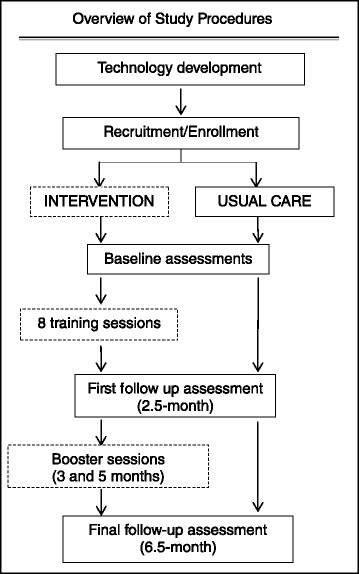
Study design and flow

See Fig. 3 for the study design and study flow and Tables 1, 2, and 3 for the data collection schedule, data collection measures, and data collection instruments, respectively. The RA, who was blinded to group assignment, conducted study assessments at baseline and at 2.5 and 6.5 months post-randomization. The NHE presented the randomized group assignment to each participant and performed the study intervention sessions. All blood samples were analyzed at the central laboratory of the academic medical center.

**Table 1:** Data collection schedule

Questionnaires/measurements	Screening visit	Baseline visit	2.5 months visit	6.5 months visit
Primary outcome measures				
Physical activity (GPAQ)		x	x	x
Diet (REAPS)		x	x	x
Medication adherence (MMAS)		x	x	x
Self-monitoring behavior		x	x	x
Secondary outcome measures				
HbA1c	x		x	x
LDL cholesterol	x		x	x
Blood pressure	x		x	x
CVD risk (FRS)		x	x	x
Quality of life (SF-12)		x	x	x
Process measures				
Knowledge/information		x	x	x
Motivation		x	x	x
Behavioral skills		x	x	x
Self-efficacy		x	x	x
Empowerment		x	x	x
Tablet usability		x	x	x
Covariates				
Patient demographics	x			
Medical comorbidity (chronic health conditions – BRFSS)		x	x	x
Social support (MOS)		x	x	x
Health literacy (s-TOFHLA)		x	x	x
Brief literacy screener (Chew)		x	x	x
Depression (PHQ-9)		x	x	x

**Table 2:** Data Collection for Outcome Measures

Outcome	Test	Measurement
Primary outcome measures	Physical activity	Measured by the 16-item Global Physical Activity Questionnaire (GPAQ) [57]
	Diet	Dietary intake assessed using the Rapid Eating and Activity Assessment for Participants Short Version (REAP-S) [58]
	Medication adherence	Measured with the 8-item self-report Morisky Medication Adherence Scale (MMAS) [59]
	Self-monitoring behavior	Measured with the Summary of Diabetes Self-Care Activities (SDSCA) scale [60]
Secondary outcome measures	HbA1c, low-density lipoprotein cholesterol (LDL-C)	Blood specimens obtained at baseline, 2.5 and 6.5 months visits
	Blood pressure (BP) measurements	An average of three BP readings done in 2-minute intervals obtained at baseline, 2.5 and 6.5 months by a trained RA using automated BP monitors (OMRON IntelliSense^TM^ HEM-907XL) with the patient seated comfortably for 5 minutes prior to the measurements
	Cardiovascular disease risk	Framingham Risk Score (FRS) estimates the 10-year risk of “hard” coronary heart disease outcomes (myocardial infarction, death) [45]
	Quality of life	The SF-12 is a valid and reliable instrument to measure functional status and will be used to assess quality of life at baseline, 2, and 6 months assessments. The SF-12 items reproduce at least 90 % of the variance in PCS-36 and MCS-36 scores [49]

**Table 3:** Data Collection for Intermediate Measures and Covariates

Measure	Data collected	Method
Process and behavioral measures	Information	Measured by the 29-item CVD Knowledge Questionnaire [61]
	Motivation	Measured by the brief Intrinsic Motivation Inventory (IMI) [62]
	Behavioral skills	Assessed with the 13-item Cardiac Self-Efficacy scale [63]
	Self-efficacy	Measured by 10-item General Self-Efficacy scale [64]
	Empowerment	Measured by 28-item Diabetes Empowerment Scale-Short Form (DES-SF) [65, 66]
	Tablet usability	Measured by the modified System Usability Scale (SUS) [67]
Covariates	Demographics	Previously validated items from the 2002 National Health Interview Survey are used to capture age, gender, race/ethnicity, marital status, household income, and health insurance
	Medical comorbidity	Medical comorbidity is documented using previously validated items on chronic health conditions from the Behavioral Risk Factor Surveillance System (BRFSS, 2010) [68]
	Social support	The Medical Outcomes Study (MOS) Social Support Survey [69] is used to measure social support
	Health literacy	The short form of the Test of Functional Health Literacy in Adults (s-TOFHLA) [70] is designed to rapidly screen patients for potential health literacy problems. The 3-item Chew Health Literacy Screening Survey will also be used [71]
	Depression	The PHQ-9 is a brief questionnaire that scores each of the 9 DSM-IV criteria for depression [72]

### Study outcomes

The primary outcome is behavioral self-management including physical activity, diet, medication adherence, and self-monitoring. The secondary outcomes are clinical measures for glycemic (HbA1c), BP, and lipid control (LDL-C); CVD risk assessed by Framingham Risk Score (FRS) [45]; and quality of life assessed by Medical Outcomes Study (MOS) Short Form (SF-12) [49]. Each outcome was measured at baseline, 2.5 months (immediate post-intervention) and 6.5 months for follow-up (post-randomization).

### Sample size determination and power analysis

Pilot studies are generally not sufficiently powered to detect statistically significant effect sizes [50]; however, small differences in primary outcome results can offer a signal of benefit and will be necessary justification that it is worth testing in a subsequent larger, adequately powered trial.

### Data analysis

#### Primary hypotheses

Compared to high-risk, low-income AAs with diabetes receiving UC, individuals randomized to the multi-component TABLETS intervention will have greater improvement in behavioral outcomes at the 6.5 months follow-up.

The intent-to-treat (ITT) sample, comprising all randomized participants, is used for primary analyses. A two-tiered analysis strategy is used for the primary and secondary aims (comparing efficacy of the TABLETS intervention compared to UC on improving the primary and secondary outcomes among high-risk AA adults). First, each primary outcome (physical activity, diet, medication adherence) and each secondary outcome (HbA1c, BP, LDL, Framingham Risk Score) is assessed separately over the 6-month study time trajectory using longitudinal data methods (generalized linear mixed effects modeling, GLMM) to compare the longitudinal trajectory of the outcomes for the two intervention groups. GLMM can accommodate different distributional assumptions for outcome variables (for example, continuous, dichotomous, or ordinal outcomes) [51] and missing data [52]. In further secondary multivariable analyses, additional covariables will be added to the model to adjust for putative confounding variables, if appropriate. Candidate covariables include, for example, age, gender, and education. Least squares means will be compared at relevant time points (2.5 and 6.5 months) using appropriate model contrasts. Second, the global benefit of the intervention based on multiple primary outcomes (physical activity, diet, medication adherence) and secondary outcomes (HbA1c, BP, LDL-C, Framingham Risk Score) will be assessed using a global statistical test (GST) [51].

An important role of a pilot study is to evaluate issues related to feasibility. Intervention feasibility measures include recruitment, compliance, and dropout proportions. For the intervention group, 95 % confidence intervals for proportions will be used to estimate the proportion of those enrolled versus those eligible, the proportion of adherers to the intervention protocol, and the proportion of drop-outs. We will describe patients’ reasons for refusing to participate and reasons for dropping out to get a better understanding of the barriers to recruitment and to implementation, respectively. Dropout proportions between the TABLETS and UC groups will be compared using logistic regression analyses.

#### Qualitative data management and analysis

In the final 6.5 months assessment, those in the intervention group will be asked to participate in 30- to 40-minute, semi-structured interviews by telephone. This is an optimal time to explore participants’ perspectives, insights, needs, and preferences in their own terms [53] as they apply to intervention components (physical activity, diet, medication adherence, self-monitoring). Just as patient-physician encounters can be examined for context cues [54], research staff will use a summary of the main context cues in the four topic areas to address what worked or did not work (useful, useless components); what enabled or delayed behavior changes (facilitators, barriers); the most helpful strategies (education, motivation, skills training) to resolve challenges; and usability and satisfaction with the tablet as a self-monitoring tool. Interview methods allow flexibility to gather additional details when needed and will yield data to refine the intervention for future implementation. With IRB approval, all interviews are audio-recorded for accuracy of data and use of selected verbatim quotes. Prior to closing, key responses are summarized, and participants are asked if they have anything to add. All data will be stored on a secure server for analysis.

Inductive data analyses will be performed to examine the natural variation among the themes, patterns, and categories that emerge from semi-structured interviews. The NVivo 10 qualitative management program used for this study is designed to systematically facilitate coding, sorting, and integrating all of the data. Two coders (including the PI) will review the transcripts and apply codes (words or brief text describing context) guided by the conceptual model and intervention components in order to provide concepts used in generating the initial line-by-line codes [55]. Triggers for important words, concepts, and comments will be noted as well as their consistency among participants. The two sets of codes will then be reviewed to determine agreement (called inter-coder reliability). Differences in interpretation of codes will be discussed and resolved between study investigators. An iterative process of coding will result in a final set of codes that can subsequently be applied to transcripts to identify themes common within and across participant interviews [55]. The goal is to address important questions that have not been examined before, to “tell a story” about the participants in this study, and to conceptualize participants’ knowledge of physical activity and diet, coping and skill-building, and ability to act on self-monitored data. Findings will help refine the intervention and emphasize elements that enhance participant uptake and motivation for sustained behavior change.

## Discussion

The study was funded in September 2013. Progress has been made in identifying and building relationships with technology companies, streamlining novel information technology processes to accommodate the multimedia aspect of the intervention (tablets, videoconferencing, home monitoring devices), refinement of intervention materials, obtaining needed study equipment (such as tablet computers with the technological capacity to execute the planned intervention), and hiring and training research personnel. The overarching goal is to develop and refine intervention processes and materials that allow tablet-based delivery of education and skills training about self-management behaviors relevant to CVD risk using real-time videoconferencing among AA adults with diabetes and other CVD risk factors. Study recruitment was initiated in September 2014 with eligibility determination, randomization, and enrollment currently underway.

The proposed study provides a unique opportunity to assess the feasibility and efficacy of a theory-driven, tablet-aided behavioral intervention that utilizes real-time videoconferencing technology for education and skills training on chronic disease self-management behaviors and CVD risk among a high-risk, low-income AA population with an uncontrolled dyad or triad of CVD risk factors. In the evolution of a paradigm shift to become more person-centered in the provision of care and to drive sustainable behavior change [56], the behavioral intervention in this pilot study is adding important value to the literature on facilitating healthier self-management behaviors. The intervention will leverage the use of a novel technology for education and skill-building that fosters improved diabetes self-management in a high-risk AA population. The findings of this study, if successful, will provide the basis for disseminating the intervention to a broader and larger sample of people, and can potentially be refined to align with clinical workflows that target a subsample of patients with poor diabetes self-management.

### Trial status

At the time of submission, the study is in the final phase of recruitment and enrollment. We have completed 92 % of the target recruitment, 63 % have completed the first (2 months) follow-up visit, and 43 % have completed the second and final (6 months) follow-up assessment.

## Abbreviations

AA: African American
BP: blood pressure
CDSM: chronic disease self-management
CONSORT: Consolidated Standards of Reporting Trials
CVD: cardiovascular disease
DSM: diabetes self-management
GLMM: generalized linear mixed effects modeling
GST: global statistical test
HbA1c: glycosylated hemoglobin A1c
HIPAA: Health Insurance Portability and Accountability Act
ICD-9: International Classification of Diseases, 9^th^ revision
IMB: Information, Motivation, and Behavioral Model
IRB: Institutional Review Board
ITT: intent to treat
LDL-C: low-density lipoprotein cholesterol
NHE: nurse health educator
NIDDK: National Institute of Diabetes and Digestive and Kidney Diseases
PCP: primary care provider
PI: principal investigator
RA: research assistant
RCT: randomized controlled trial
SC: South Carolina
SD: standard deviation
T2DM: type 2 diabetes mellitus
TABLETS: Tablet-Aided BehavioraL intervention EffecT on Self-management skills
UC: usual care
